# Ultrasonic Phased Array Sparse-TFM Imaging Based on Sparse Array Optimization and New Edge-Directed Interpolation

**DOI:** 10.3390/s18061830

**Published:** 2018-06-05

**Authors:** Hongwei Hu, Jian Du, Chengbao Ye, Xiongbing Li

**Affiliations:** 1 College of Automotive and Mechanical Engineering, Changsha University of Science and Technology, Changsha 410114, China; hhwlucky@163.com (H.H.); dujian1009@foxmail.com (J.D.); 15507482549@163.com (C.Y.); 2 School of Traffic and Transportation Engineering, Central South University, Changsha 410075, China

**Keywords:** ultrasonic phased array, sparse-TFM imaging, sparse array optimization, new edge-directed interpolation

## Abstract

The ultrasonic phased array total focusing method (TFM) has the advantages of full-range dynamic focusing and high imaging resolution, but the problem of long imaging time limits its practically industrial applications. To reduce the imaging calculation demand of TFM, the locations of active array elements in the sparse array are optimized by combining almost different sets with the genetic algorithm (ADSGA), and corrected based on the consistency of the effective aperture with the equivalent point diffusion function. At the same time, to further increase the imaging efficiency, a sparse-TFM image with lower resolution is obtained by reducing the number of focus points and then interpolated by the new edge-directed interpolation algorithm (NEDI) to obtain a high quality sparse-TFM image. Compared with TFM, the experimental results show that the quantitative accuracy of the proposed method is only decreased by 1.09% when the number of sparse transmitting elements reaches 8 for a 32-element transducer, and the imaging speed is improved by about 16 times with the same final pixel resolution.

## 1. Introduction

Ultrasonic phased array testing has the advantages of high sensitivity and favorable adaptability to complex components, and has been widely used in nondestructive testing for key equipment [1,2,3]. Holmes et al. [4] firstly proposed the concepts of full matrix capture (FMC) and the full matrix data model, and established the total focusing method (TFM) algorithm using FMC. Compared with conventional ultrasonic phased array testing, TFM can realize the focus of any points within the measured area by post-processing the full matrix data of the transducer array [5,6,7]. This method has therefore been increasingly used in the fields of aviation, nuclear power, composite materials and so on [8,9,10].

However, due to the large amount of full matrix data involved, TFM imaging calculations are time-consuming, which limits its applications in some industrial fields, especially those with real-time requirements. To solve this problem, the computing time can be reduced by improving the hardware architecture using a GPU [11] or parallel computing with multiple Field-Programmable Gate Arrays (multi-FPGA) [12], but then the cost will increase significantly. Another important research topic is how to improve the computational efficiency of the imaging algorithm while maintaining the image quality. Since the full matrix data are redundant [13], the sparse array design technology can be used to reduce the computing data for post-processing. In order to reduce the artifacts (caused by the physical properties of the ultrasonic wave such as direction, reflection, refraction and penetration) in phased array imaging and to improve the imaging efficiency, the element layout of the sparse array can be optimized using the genetic algorithm (GA) [14] or the simulated annealing algorithm [15]; these are random optimization algorithms, but both are effective only for the optimization of a small sparse array. Moreover, the optimized array has poor consistency in sound field characteristics, the calculated value of the peak of side-lobe (*PSL*) and main-lobe width (*MLW*) are different in each time. Oliveri et al. [16] firstly applied almost different sets (ADS) to the design of a sparse array, in which the element layout can be determined analytically and where the computational complexity is much lower than that of the random optimization algorithms; however, it is difficult to obtain a sparse array with an arbitrary array aperture and thinning factors. Based on the above studies, a method called ADSGA, which combines ADS and GA, is applied in the design of the radar sparse array [17]. Compared with the ADS method, the ADSGA method can obtain the sparse array with better side lobe performance using fewer iteration times, and can obtain an arbitrary array aperture and sparseness. However, this method ignores the variation of the emitted sound field of the array after sparsing, which leads to significantly different effective apertures between the sparse array and the full array and can affect the imaging performance of the sparse array.

In order to further improve the computational efficiency, fewer imaging points can be chosen for imaging. To improve the resolution, an image with fewer imaging points should be interpolated. Traditional interpolation algorithms include nearest neighbor interpolation [18], bilinear interpolation [19] and cubic convolution interpolation [20]. In most cases, these interpolation methods use the same interpolation functions and do not take into account the influence of gray value mutation of the edge pixel in image interpolation; hence, they produce interpolated images with blurred edges and blocking effects [21]. Wei [22] proposed a contrast-guided image interpolation (CGI) algorithm which not only protected the pixels on the edge, but also the non-edge pixels within a certain range from the edge. The image quality was significantly improved, but had a higher computational complexity. A new edge-directed interpolation algorithm (NEDI) was proposed by Li [23], based on the geometric duality between the low-resolution covariance and the high-resolution covariance. Adaptive interpolation of the image was conducted, and was shown to have a lower computational complexity than current methods; hence, it is fairly attractive for real-time image applications and offers improved visual quality of the interpolated images.

In this paper, a sparse-TFM imaging method based on sparse array optimization and NEDI is developed to improve inspection efficiency. The sparse array is first optimized by combining ADS with the GA in an ADSGA. The sound field characteristics of the sparse array are modified under the conditions of the same effective aperture with the full matrix, and a modified sparse-TFM imaging model based on the ADSGA method is established. Secondly, fewer imaging points are selected to further improve the computational efficiency and the low resolution sparse-TFM image is interpolated using the NEDI method to improve the imaging quality. Finally, the proposed sparse-TFM is applied to the testing of a steel specimen with some circular arc distributed side drilled holes (SDHs), and the imaging quality and computational efficiency are analyzed quantitatively.

## 2. Materials and Methods

According to the principle of the TFM algorithm, each element in the phased array transducer with N elements is excited in turn, and all elements in the transducer receive echo signals until the Nth element is excited. Saving each received data as $e_{11}(t)$, $e_{12}(t)$,…, $e_{1N}(t)$, $e_{21}(t)$, $e_{22}(t)$,…$e_{2N}(t)$…$e_{NN}(t)$, the FMC is obtained. TFM is the post-processing imaging method using all the information of this full matrix data. 

**Figure 1:**
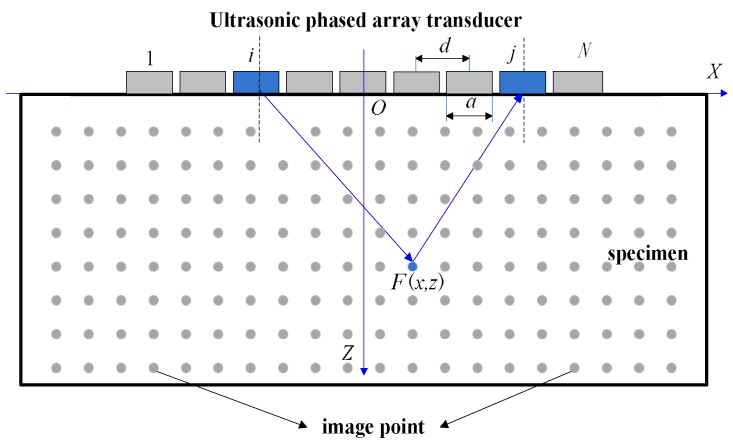
Schematic diagram of TFM imaging.

A one-dimensional linear phased array transducer is placed on the surface of the specimen, and the established coordinate system OXZ is shown in Figure 1. The origin O is located in the array center of the phased array transducers, the X axis is directed to the right along the array direction, and the Z axis is perpendicular to the array direction and points to the measured area. For each focus point (*x*, *z*), the ultrasonic echo signals of all transmit-receive pairs are superimposed at that point, and the intensity of the image (that is the grayscale value of the pixels) *I*(*x*, *z*) is obtained and expressed as [4]:

(1) $$I(x,z)=\sum_{i=1}^{N}\sum_{j=1}^{N}e_{ij}\left(\frac{\sqrt{{\left(x_{tx}-x\right)}^{2}+{\left(z_{tx}-z\right)}^{2}}+\sqrt{{\left(x_{rx}-x\right)}^{2}+{\left(z_{rx}-z\right)}^{2}}}{c}\right)$$

where (*x_tx_*, *z_t__x_*) is the coordinate of the transmitting element, (*x_rx_*, *z_rx_*) is the coordinate of the receiving element, *N* is the number of elements in the transducer, $e_{ij}()$ is the signal that transmitting from element *i* and receiving in element *j*, *c* is the speed of sound in the specimen.

From Equation (1), it can be seen that the post-processing of TFM requires a large number of calculations, especially for a large element array. This problem can be solved by reducing the number of active transmitting or receiving elements and the amount of data collected by the FMC [24].

**Figure 2:**
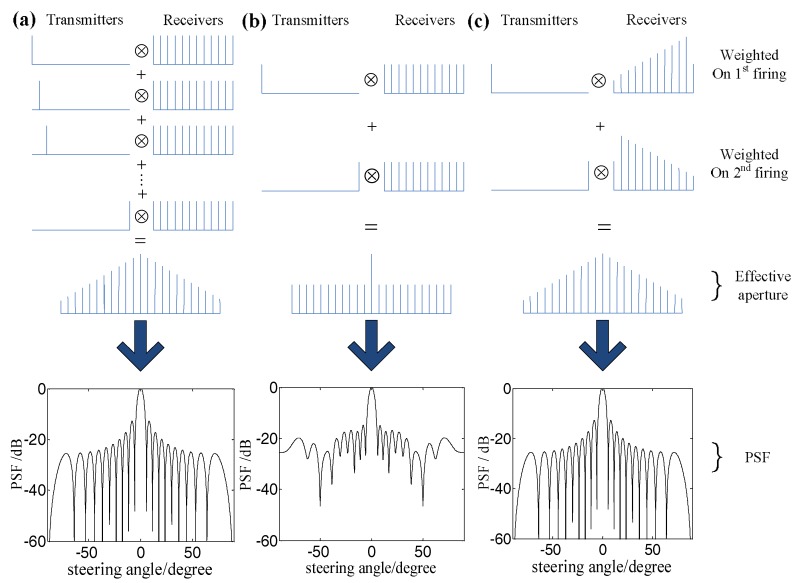
The array effective aperture and point spread function under different methods: (**a**) TFM; (**b**) sparse-TFM; and (**c**) corrected sparse-TFM.

Moreover, in order to make the sound field characteristics of the sparse array consistent with those of the full array, the sparse transmit/receive elements should be weighted to make the sparse array coincide with the effective aperture of the full array by selecting the appropriate weight functions [24]. Figure 2a–c show the effective aperture and the point spread functions (PSF) in the far field [24] of TFM, the uncorrected sparse-TFM and the corrected sparse-TFM, respectively. It can be seen that the corrected sparse array has the same effective aperture with the full array, and the intensity of each point in the corrected sparse-TFM image can be expressed as [25]:

(2) $$I_{C}(x,z)=\sum_{i=1}^{N_{T}}\sum_{j=1}^{N_{R}}\omega_{T}^{i}\omega_{R}^{j}e_{i,j}\left(\frac{\sqrt{{\left(x_{tx}-x\right)}^{2}+{\left(z_{tx}-z\right)}^{2}}+\sqrt{{\left(x_{rx}-x\right)}^{2}+{\left(z_{rx}-z\right)}^{2}}}{c}\right)$$

where *N*_T_ and *N*_R_ are the number of transmitting and receiving elements in the sparse array respectively, $\omega_{T}$ and $\omega_{R}$ are the weight functions of the transmitting and receiving array respectively.

## 3. ADSGA-Based Sparse Array Optimization

**Figure 3:**
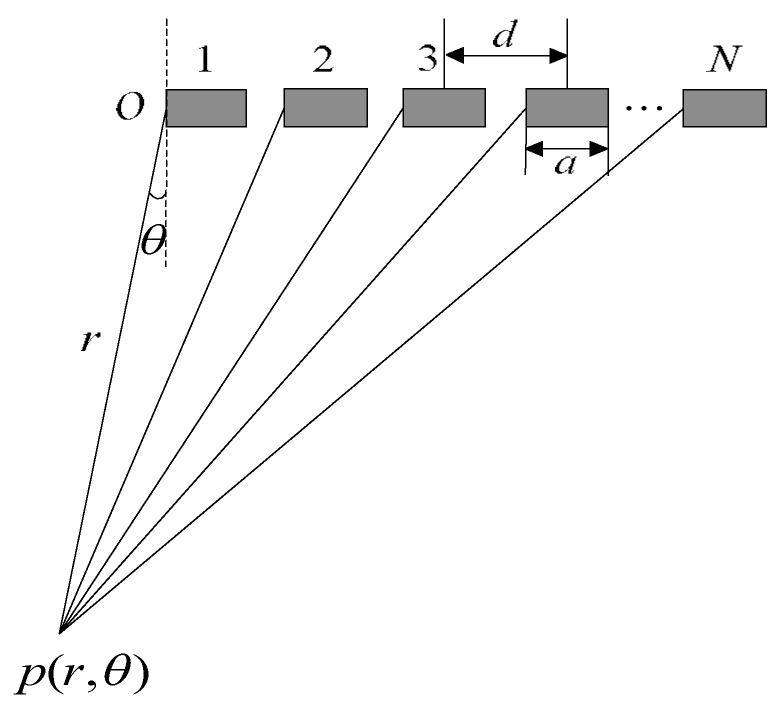
Schematic diagram of the sound pressure calculation of the phased array transducer.

The location of active elements in the sparse array is very important for imaging performance. To improve the image quality with sparse array, the location of each active element in the sparse array is optimized using ADSGA. *N* is the total number of elements of the target sparse array, *K* is the number of active elements, *a* is the width of each element, and *d* is the pitch of the elements. The optimization aims to minimize *PSL* and *MLW* of the sparse array. For the linear array shown in Figure 3, the sound pressure of the single element *i* can be defined as follows [25]:

(3) $$\xi_{i}(r,\theta ,t)={\left(\frac{\xi_{0}}{r}\right)}^{1/2}\frac{\sin(ka\sin\theta /2)}{k\sin\theta /2}\exp(-\frac{jka\sin\theta }{2})\exp\left[j\left(\omega t-kr\right)\right]$$

where *r* and θ are the distance and the direction angle between the imaging point and the array element respectively, *k* is the wave number, and ω is the angular frequency.

The synthesized sound pressure ξ(r,θ,t) and the beam directivity function H(θ) can be defined as follows:

(4) $$\xi (r,\theta ,t)=\sum_{i=1}^{N}g_{i}\xi_{i}(r,\theta ,t)$$

(5) $$H(\theta )=\left|\frac{\xi (r,\theta ,t)}{\xi (r,\theta_{s},t)}\right|$$

where $g_{i}$ is the binary coefficient, $g_{i}$ is 1 when the element *i* is active, and $g_{i}$ is 0 when it is inactive, $\theta_{s}$ is the steering angle of the phased array, which is set to 0 here. According the figure of the beam directivity obtained by the H(θ) in Equation (5), the *PSL* and *MLW* can be obtained.

The key steps in sparse array design using the ADSGA method are shown as follows:

Step 1. Population initialization. According to the parameters *N* and *K* of the sparse array, the almost differential sets of relevant parameters (*N*, *K*, Λ, *t*)-ADS and the corresponding binary sequences are selected from the ADS library [26,27], where Λ and *t* are the characteristic parameters of the ADS, and are the number of non-zero elements in the *N* order Abelian group and the number of non-zero elements appearing in the compound set, respectively. The *N*-1 shift types of the binary sequence are obtained by cyclic shift and ranked based on the *PSL* values of corresponding binary sequences in these *N* ADS [28]. The top-ranked *P*/2 individuals are chosen as the initial population (*P* is the total number of individuals in each generation during the iteration):

(6) $$\begin{array}{l} \rho_{p}=\left\{b_{p}\left(n\right)=w^{\left(p\right)}\left(n\right);n=0,\ldots ,N-1\right\}\\ 1\leq p\leq P/2 \end{array}$$

where *p* is the serial number of the individual in each generation, $\rho_{p}$ is the binary sequence of the *p*-th individual, *n* is the digits of the binary sequence, $w^{\left(p\right)}\left(n\right)$ is the value of the *n*-th digit in the binary sequence of the *p*-th ADS. If $n\in D^{\left(\sigma \right)}$, which means that the number of cyclic shift is σ and *n* is the element of the ADS shift types $D^{\left(\sigma \right)}$, $w^{\left(p\right)}\left(n\right)$ = 1; otherwise, $w^{\left(p\right)}\left(n\right)$ = 0. $b_{P}\left(n\right)$ is the gene values of the *n*th individual in the *p*-th population. $b_{P}\left(n\right)=1$ when the element *n* is active, $b_{P}\left(n\right)=0$ when the element n is inactive. The remaining *P*/2 initial individuals are generated randomly:

(7) $$\rho_{p}=\left\{b_{p}\left(n\right)=r_{p}\left(n\right);n=0,\ldots ,N-1\right\},\;\;P/2\leq p\leq P$$

where $r_{p}\left(n\right)$ is 0 or 1 randomly.

Step 2. Fitness evaluation. The fitness function used here is expressed as: *Fit* = *ψ*_1_*PSL + *ψ*_2_*MSL, where *ψ*_1_ and *ψ*_2_ are the coefficient values selected according to different optimization targets. The characteristics of the main lobe and the side lobe are needed to be optimized simultaneously, so both *ψ*_1_ and *ψ*_2_ are set to 1.

Step 3. Selection, crossover and mutation. The initial group *G*(*n*) is ranked according to the fitness function value, and the inferior half chromosomes are discarded. Presetting a crossover probability and pairing the remaining *M*/2 individuals, some genes in each combination are replaced according to this probability, and then the new *M*/2 individuals are generated. The number of individuals in the group will still be *M* after this processing. Moreover, according to the mutation probability, the genes of the individuals in the population are replaced by the corresponding alleles, which means that the gene values change from 0 to 1 or from 1 to 0. *v* is the thinning factors, *b*(*U*) and *b*(*V*) are the gene values of the *U*th and *V*th individual in the population respectively, then the bit mutation probability is defined as follows [17]:

(8) $$H_{BM}\left(n\right)=\frac{\left[N\times v-\sum_{U=0}^{n-1}b\left(U\right)\right]}{N-V}\times \left[1-2b\left(V\right)\right]+b\left(V\right)$$

Step 4. Steps 2 and 3 are repeated until the preset number of iterations is reached. The optimal chromosomes in each circulation are recorded and transferred to the corresponding array structure, then the optimal sparse array can be obtained.

**Figure 4:**
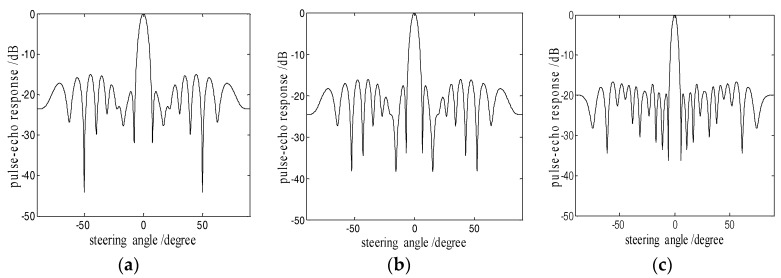
Directivity diagrams of the 8-element sparse arrays: (**a**) uniform; (**b**) GA; and (**c**) ADSGA.

Taking a 32-element array transducer as an example, the ADSGA optimization of sparse arrays is carried out. The thinning factors *v* is set to 0.25, which means the number of active elements *K* is 8. The number of the initial population group is set to 40, the number of iterations is set to 400, the crossover probability is set to 0.8, and the mutation probability can be calculated by Equation (8). Figure 4a is the directivity diagram of the 8-element uniform sparse array, and Figure 4b,c are the directivity diagrams of 8-element sparse array optimized using GA and ADSGA respectively. Table 1 lists the performance of the main lobe and the side lobe, one can see that the sparse array optimized using ADSGA has better imaging characteristics with a narrower *MLW* and smaller *PSL* in comparison with the uniform sparse array and sparse array optimized using GA.

## 4. Sparse-TFM Based on NEDI

To further improve the imaging efficiency, a low resolution (LR) image $I_{\mathrm{LR}}(x,z)$ can be obtained in advance by increasing the pixel distance in the measured area according to Equation (2). Then the obtained image $I_{\mathrm{LR}}(x,z)$ is interpolated using the NEDI method. The basic idea of NEDI is that the value of each pixel point in the high resolution (HR) image is obtained by calculating the local covariance coefficient of each pixel point in the LR image according to the geometric duality of the covariance in the HR and LR image.

**Figure 5:**
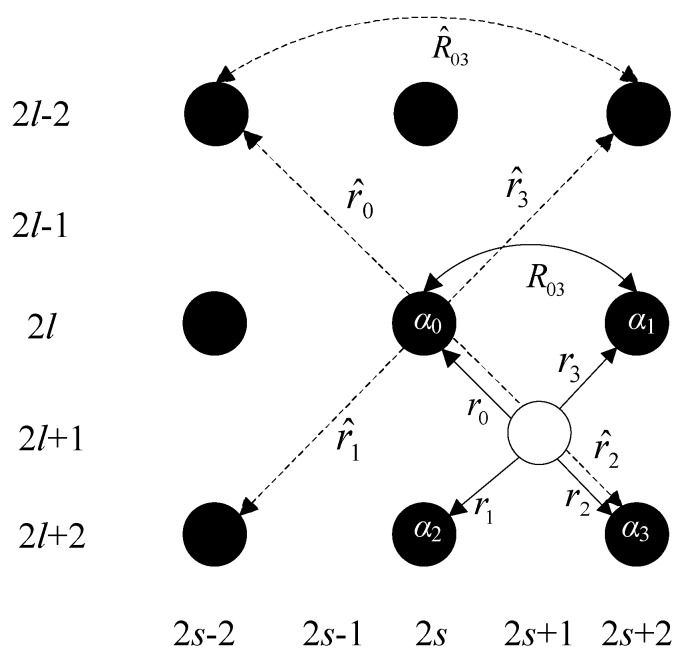
The first stage of interpolation.

If the number of pixels in the sparse-TFM image $I_{\mathrm{LR}}(l,s)$ is *L* × *S* (*l* and *s* respectively are the serial numbers of the row and column of the imaging point, *L* and *S* are the number of rows and columns of image respectively), the number of pixels of the corresponding HR image $I_{\mathrm{HR}}(2l,2s)$ is 2*L* × 2*S*. The original imaging points remain the same during the interpolation, namely $I_{\mathrm{HR}}(2l,2s)\;=\;I_{\mathrm{LR}}(l,s)$. The interpolation is divided into two steps. In the first step, the intensity of the central point is obtained according to the interpolation of four original imaging points. As shown in Figure 5, the black dots represent the known imaging points, and the white dots represent the points to be interpolated. The intensity of the interpolation point (2*l* + 1, 2*s* + 1) is obtained as [29]:

(9) $$I(2l+1,2s+1)=\sum_{\gamma =0}^{1}\sum_{q=0}^{1}\alpha_{2\gamma +q}I(2(l+\gamma ),2(s+q))$$

where I(2(l+γ),2(s+q)) is the intensity of four imaging points nearest to the points to be interpolated; γ and *q* respectively are the lateral and longitudinal offset points of the interpolation point from the center point (*l,s*); $\alpha_{2\gamma +q}$ is the interpolation weight coefficient. Imaging intensity I(2(l+γ),2(s+q)) is known, so I(2l+1,2s+1) can be obtained after calculating the interpolation weight coefficient $\alpha_{2\gamma +q}$.

According to the classical Wiener filtering theory, the optimal linear interpolation coefficient of minimum mean squared error (MMSE) can be expressed as [30]:

(10) $$\alpha =R^{-1}r$$

where $R=[R_{\beta \phi }]$ and $r=[r_{\beta }]$ are the local covariance coefficients in the HR image, β∈{0,1,2,3},ϕ∈{0,1,2,3}. It can be seen from Figure 5 that the covariance coefficients $R_{\beta \phi }$ and $r_{\beta }$ in HR image and ${\hat{R}}_{\beta \phi }$ and ${\hat{r}}_{\beta }$ in LR image have geometric duality. $R_{\beta \phi }$ and ${\hat{R}}_{\beta \phi }$ respectively connect the same pair of imaging point in the same direction at different resolutions, as well as $r_{\beta }$ and ${\hat{r}}_{\beta }$. Thus, $R_{\beta \phi }$ and $r_{\beta }$ can be obtained by calculating ${\hat{R}}_{\beta \phi }$ and ${\hat{r}}_{\beta }$. The standard covariance method can be used to calculate the value of ${\hat{R}}_{\beta \phi }$ and ${\hat{r}}_{\beta }$ in a local window of *M* × *M*:

(11) $$\hat{R}=\frac{1}{M^{2}}CC^{T},\hat{r}=\frac{1}{M^{2}}Cy$$

where $y={\left[y_{1},y_{2}\cdots y_{\mu },\cdots y_{M^{2}}\right]}^{T}$ is a vector composed of *M* × *M* pixel points in local window *W*, *C* is a numerical matrix of 4 × *M*^2^, and the μth column vector is the intensity of the nearest four points in the diagonal direction of $y_{\mu }$. According to Equations (10) and (11), it can be derived that:

(12) $$\alpha =(CC^{T})-Cy$$

Based on Equation (12), the optimal interpolation weight coefficient α of I(2l+1,2s+1) is calculated, and the intensity I(2l+1,2s+1) can then be obtained by substituting α into Equation (9).

**Figure 6:**
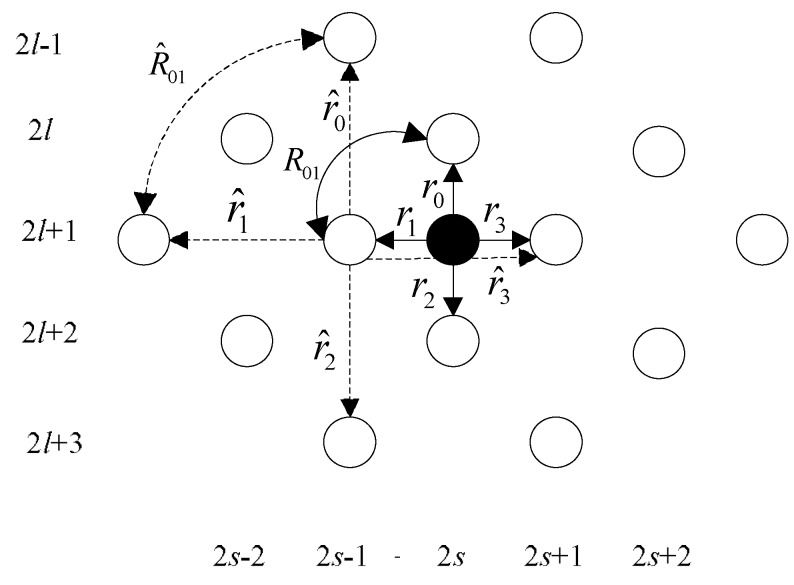
The second stage of interpolation.

In the second step, the interpolation is conducted between the known pixel points and the pixel points obtained in the first step. As shown in Figure 6, the white dots represent the known pixel points and the black dots represent the interpolation points. Since the four points in the diagonal direction nearest to the black dots are not points on the LR image, Equation (9) does not work for the interpolation here. However, the interpolation of the points can be obtained using the points on the LR image and the estimated pixel points [29]:

(13) $$I(2l+1,2s)=\alpha_{0}I(2l,2s)+\alpha_{1}I(2l+1,2s-1)+\alpha_{2}I(2l+2,2s)+\alpha_{3}I(2l+1,2s+1)$$

In the same way, the interpolation weight coefficient α can be obtained and the intensity of the interpolation points is finally obtained.

## 5. Experiments

**Figure 7:**
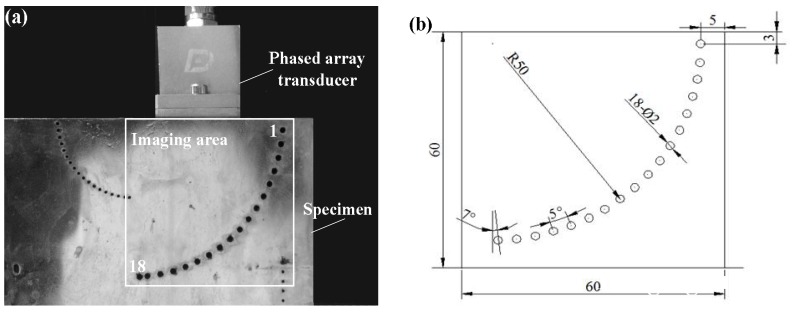
(**a**) Diagram of the experiment; (**b**) specimen in the ultrasonic phased array detection experiment.

As shown in Figure 7, a steel specimen including SDHs is used for the experiments. There are 18 SDHs with diameter of 2 mm in the detection area; these are numbered 1–18 from top to bottom, and their geometric sizes are shown in Figure 7b. The detection area is 60 mm × 60 mm. The 5L32-0.6 × 10-type ultrasonic phased array transducer (Guangzhou Doppler Electronic Technology Co., Ltd., Guangzhou, China) was used, with a total number of elements of 32, a pitch between each element of 0.6 mm, an element width of 0.5 mm and a center frequency of 5 MHz. The sound velocity in the specimen was measured as 5900 m/s. The full matrix data is collected and stored using the FMC method at the sampling frequency of 100 MHz. The experimental calculation was carried out on a computer with a Core i5 CPU and a 4 GB RAM.

**Figure 8:**
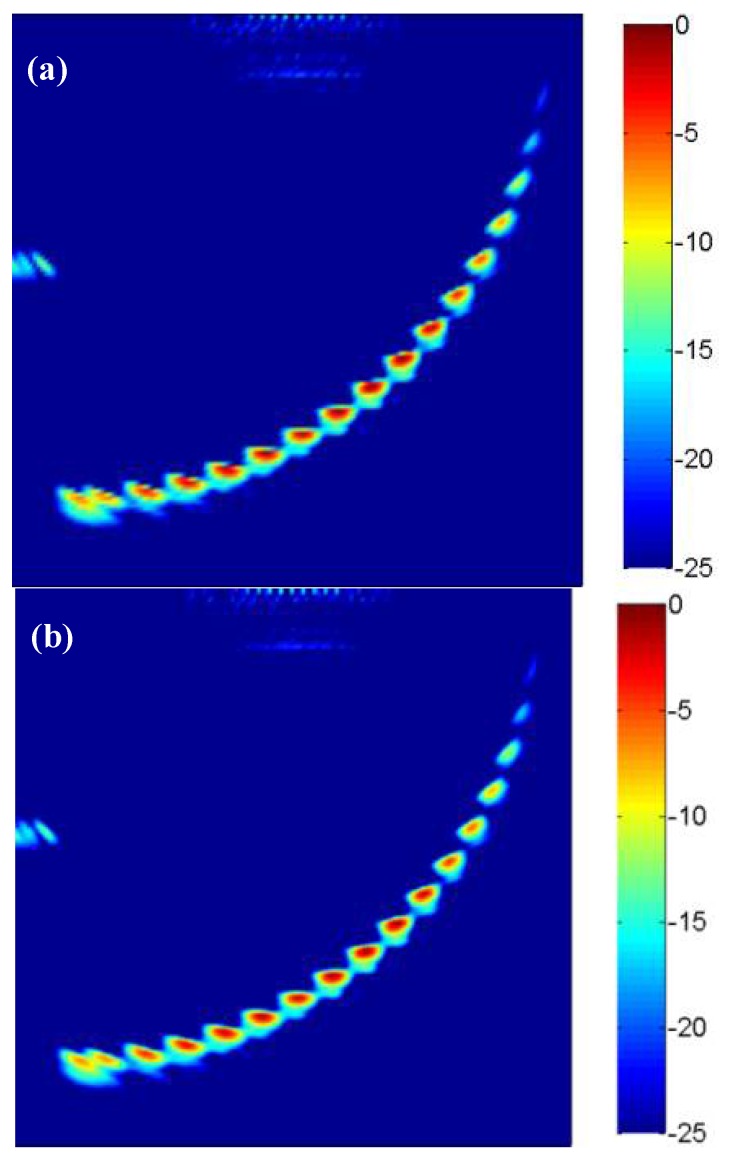

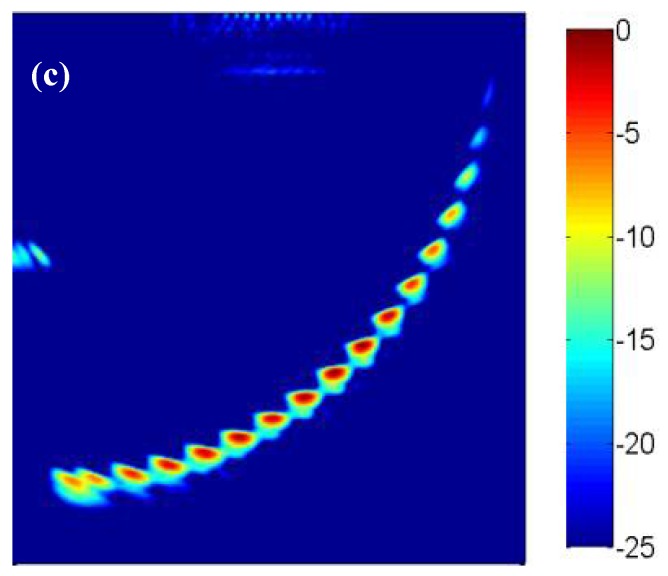
The sparse total focus imaging (**a**) No interpolation; (**b**) Bilinear interpolation; (**c**) NEDI interpolation with 8-element.

The lateral and longitudinal pixel distances were both set as 0.4 mm and the imaging area was divided into a grid of 151 × 151 pixels. First, the sparse array obtained by ADSGA method was used for the sparse-TFM and corrected using the effective aperture method. The imaging results are shown in Figure 8a. Then the 2 × 2 bilinear interpolation [19] and NEDI interpolation are performed on the Figure 8a, and the results of the interpolation are shown in Figure 8b,c, respectively. As shown in Figure 8, the edge of defect holes is blurred in the image without interpolation, and a certain amount of key pixel information is missing due to the large pixel distance, which is harmful to the quantitative analysis of defect holes. In contrast, in the image with interpolation, the edge of the defect holes is smooth.

**Figure 9:**
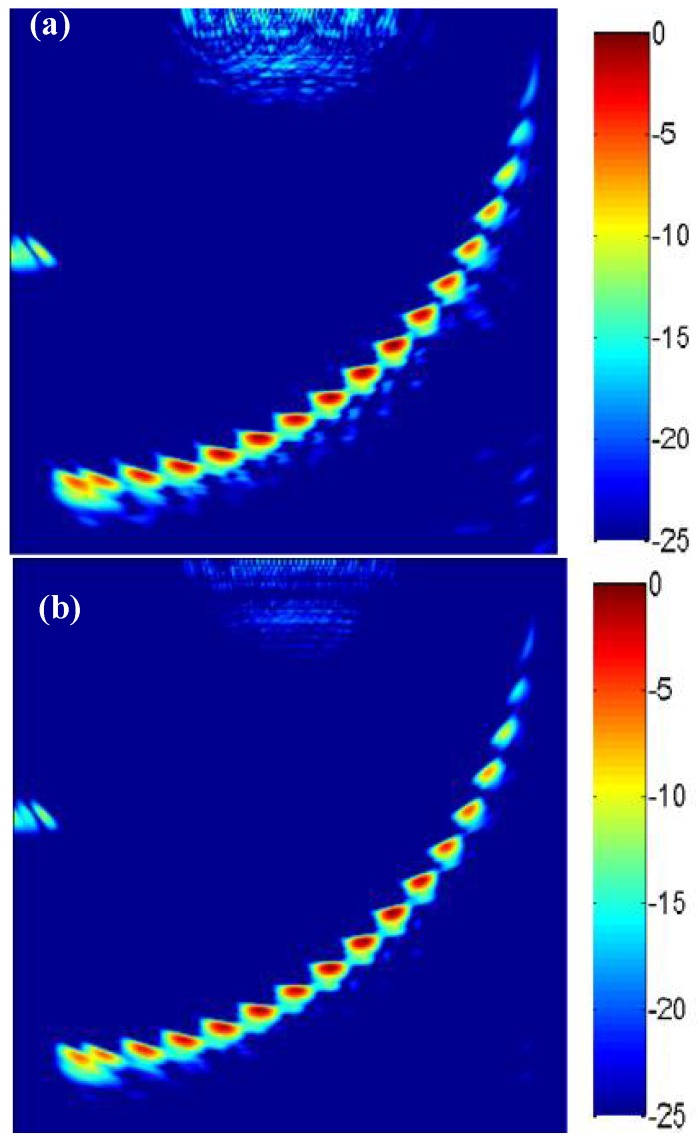

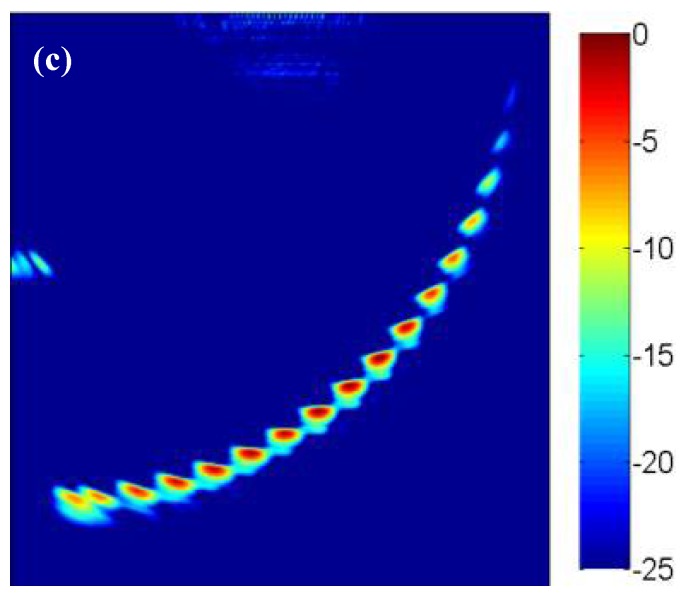
The results with different imaging methods (**a**) ADSGA sparse-TFM; (**b**) ADSGA-NEDI sparse-TFM; (**c**) TFM.

Then the lateral and longitudinal pixel distances are set as 0.2 mm and the imaging pixel is divided into 301 × 301. The collected full matrix data are used for the TFM imaging and sparse-TFM imaging. The sparse-TFM imaging based on ADSGA (ADSGA sparse-TFM), the corrected sparse-TFM imaging based on ADSGA-NEDI method (ADSGA-NEDI sparse-TFM) and the TFM imaging are shown in Figure 9. Compared with the 8-element sparse-TFM imaging based on ADSGA method, one can see that the artifacts and noise in the corrected sparse-TFM imaging based on ADSGA-NEDI method proposed in this paper are greatly reduced, which is almost the same with the TFM imaging.

## 6. Discussion

**Figure 10:**
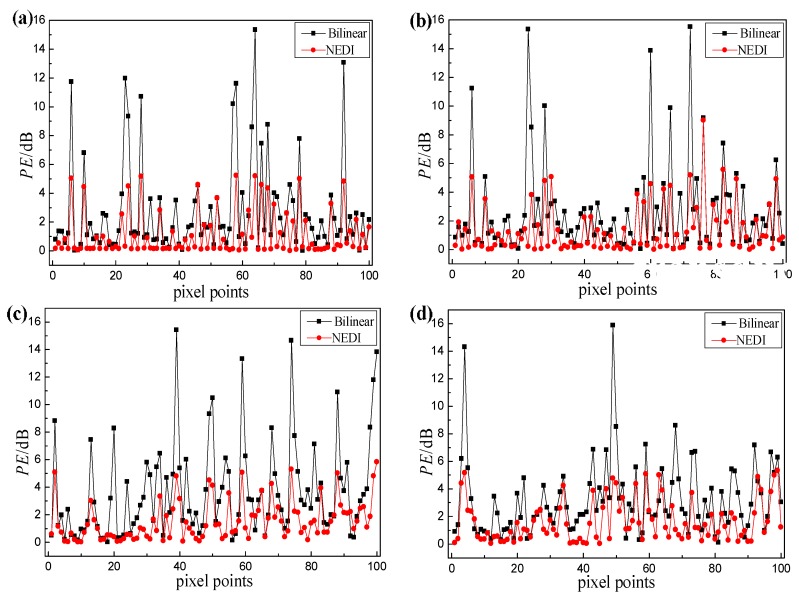
Pixel error analysis of centergrid of different holes (**a**) hole 5; (**b**) hole 7; (**c**) hole 9; (**d**) hole 11.

Pixel error (*PE*) and mean pixel error (*MPE*) are introduced here as evaluation indices for a quantitative comparison of the images with and without interpolation, and can be expressed as follows:

(14) $$PE(i)=\left|I(i)-I_{\mathrm{TFM}}(i)\right|,(i=1,2,\cdots ,m)$$

(15) $$MPE(i)=\frac{1}{M}\sum_{i=1}^{M}\left|I(i)-I_{\mathrm{TFM}}(i)\right|,(i=1,2,\cdots ,m)$$

where *m* is the total number of pixel points in the imaging area, $I_{\mathrm{TFM}}(i)$ is the intensity of each point in the TFM image, *I*(*i*) is the intensity of each point after interpolation. The central grid of 10 × 10 pixels including defect holes 5, 7, 9 and 11 is selected as the detection area, and the error values of the interpolated images around this four defect holes with respect to the TFM images are compared. The results are shown in Figure 10.

As Figure 10 shows, the *PE* values in sparse-TFM imaging based on the NEDI method (NEDI sparse-TFM) regarding the TFM imaging are within 5.5 dB, except for a few points. Moreover, the *PE* values of most points are within 2 dB, and these areas can be reconstructed well using interpolation. In addition, the *MPE* values of the points around this four defect holes are 1.11 dB, 1.34 dB, 1.54 dB and 1.62 dB respectively; these are reduced by 1.28–2.13 dB in comparison with traditional bilinear interpolation. It can be seen that the imaging results for the proposed NEDI sparse-TFM imaging are closer to those from TFM. 

**Figure 11:**
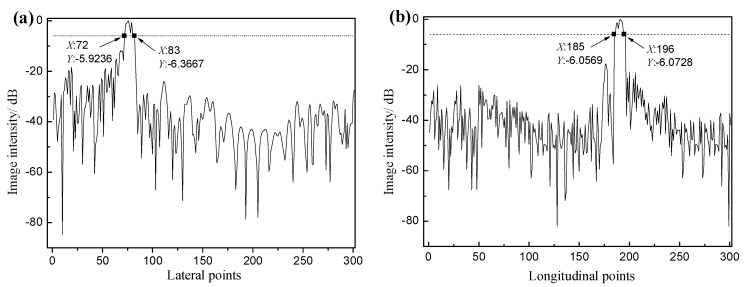
(**a**) Lateral and (**b**) longitudinal sound pressure distribution of defect hole 9.

In order to further analyze the absolute precision error of the image, we take the defect hole 9 as an example. The imaging intensity of the defect hole along the lateral and longitudinal center axes in Figure 9b is extracted and normalized. Then the normalized image intensity is expressed in decibels as shown in Figure 11, and the lateral and longitudinal size of the defect holes are measured using the 6-dB-drop method. The evaluated lateral and longitudinal sizes of hole 9 are both 2.2 mm and the error value is only one pixel distance, which is mainly caused by the influence of the number of transducers, the sound speed error and the pixel distance.

Similarly, to compare the quality of images in different methods in Figure 9, the area of each defect hole is measured using the 6-dB-drop method. In view of the large angular deflection effect on the imaging of hole 1, only holes 2–18 are analyzed. The evaluated area and quantitative error of these defect holes are shown in Table 2. As can be seen from Table 2, the average errors of the TFM image, the ADSGA sparse-TFM image and the ADSGA-NEDI sparse-TFM image proposed in this paper are 4.98%, 9.84% and 6.07%, respectively. It can be seen that the average measurement accuracy of ADSGA-NEDI sparse-TFM is improved by 3.77% in comparison with ADSGA sparse-TFM, and which is only 1.09% lower than the average measurement accuracy of TFM. Furthermore, the imaging time of TFM, ADSGA sparse-TFM and ADSGA-NEDI sparse-TFM are respectively 17.2 min, 4.3 min and 1.08 min. Compared with TFM, the imaging speed of ADSGA-NEDI sparse-TFM is improved by about 16 times.

## 7. Conclusions

A corrected sparse-TFM imaging method based on sparse array optimization and NEDI is proposed in this paper, and the imaging quality and computational efficiency are analyzed quantitatively. The following conclusions can be drawn:

(1) Compared with the uniform sparse array and sparse array optimized using GA, the sparse array optimized by ADSGA has a narrower *MLW* and a smaller *PSL*. In this study, the acoustic field of the sparse array is modified, and the modified sparse total focusing imaging model based on ADSGA method is developed.
(2) The low resolution sparse TFM image is interpolated using the NEDI method to improve the imaging resolution. The *PE* of each pixel point between the image in this method and TFM is within 5.5 dB, and the *MPE* of each imaging area is within 1.62 dB, which has an advantage of 1.28–2.13 dB over the traditional bilinear interpolation method.
(3) Compared with the ADSGA sparse-TFM, the average measurement accuracy of ADSGA-NEDI sparse-TFM is improved by 3.77%. In addition, compared with TFM, the average error of ADSGA-NEDI sparse-TFM is increased by only 1.09% when the sparse transmitting elements number reaches 8 for a 32-element transducer, while the imaging speed is improved by about 16 times.

The proposed method can greatly improve the computational efficiency of TFM imaging method with good imaging quality and provide an important reference for the industrial application of ultrasonic phased array TFM imaging. With the development of image interpolation technology and the application of parallel computing in the TFM, the higher-quality images will be obtained in a shorter time. Our future work will focus on the sparse-TFM imaging for curved components.

## Figures and Tables

**Table 1:** Performance of the 8-element sparse arrays.

Sparse Type	Array Layout	*MLW* (°)	*PSL* (dB)
Uniform	10001000100010001000100010001000	10.23	−12.27
GA	00000110000100001001100000011000	8.0	−16.13
ADSGA	10000000000110011100010000000001	6.3	−17.55

**Table 2:** Measurement results of the defect holes using different methods.

No. of SDH	Evaluated Area/mm^2^			Deviation Relative to the Actual Value (%)		
	ADSGA Sparse-TFM	ADSGA-NEDI Sparse-TFM	TFM	ADSGA Sparse-TFM	ADSGA-NEDI Sparse-TFM	TFM
2	2.9	2.86	2.83	7.64	8.92	9.87
3	2.9	2.95	2.93	7.64	6.05	6.69
4	2.99	3	3.05	4.78	4.46	2.87
5	2.97	3.06	3.12	5.41	2.54	0.64
6	3	3.09	3.09	4.46	1.59	1.59
7	2.9	2.97	3.11	7.64	5.41	0.96
8	3.31	3.19	3.2	5.41	1.60	1.91
9	3.55	3.43	3.31	13.06	9.24	5.41
10	3.49	3.37	3.35	11.15	7.32	6.69
11	3.61	3.42	3.38	14.97	8.91	7.64
12	3.57	3.39	3.27	13.70	7.96	4.14
13	3.51	3.3	3.34	11.78	5.10	6.37
14	3.48	3.29	3.38	10.83	4.78	7.64
15	3.44	3.37	3.29	9.55	7.32	4.78
16	3.54	3.29	3.25	12.74	4.78	3.50
17	3.58	3.31	3.31	14.01	5.41	5.41
18	3.53	3.51	3.41	12.42	11.78	8.60
